# Identification of candidate genomic regions for chicken egg number traits based on genome-wide association study

**DOI:** 10.1186/s12864-021-07755-3

**Published:** 2021-08-10

**Authors:** Xiurong Zhao, Changsheng Nie, Jinxin Zhang, Xinghua Li, Tao Zhu, Zi Guan, Yu Chen, Liang Wang, Xue Ze Lv, Weifang Yang, Yaxiong Jia, Zhonghua Ning, Haiying Li, Changqing Qu, Huie Wang, Lujiang Qu

**Affiliations:** 1 Department of Animal Genetics and Breeding, State Key Laboratory of Animal Nutrition, National Engineering Laboratory for Animal Breeding, College of Animal Science and Technology, China Agricultural University, Beijing, 100193 China; 2 Beijing Municipal General Station of Animal Science, Beijing, 100107 China; 3 Institute of Animal Sciences, Chinese Academy of Agricultural Sciences, Beijing, 100193 China; 4 College of Animal Science, Xinjiang Agricultural University, Urumqi, 830000 China; 5 Engineering Technology Research Center of Anti-aging Chinese Herbal Medicine of Anhui Province, Fuyang Normal University, Fuyang, 236037 Anhui China; 6 College of Animal Science, Tarim University, Alar, 843300 Xingjiang China; 7 Key Laboratory of Tarim Animal Husbandry Science and Technology, Xinjiang Production & amp; Construction Corps, Alar, 843300 Xingjiang China

**Keywords:** Chicken, Egg number, GWAS, SNPs, Genomic region

## Abstract

**Background:**

Since the domestication of chicken, various breeds have been developed for food production, entertainment, and so on. Compared to indigenous chicken breeds which generally do not show elite production performance, commercial breeds or lines are selected intensely for meat or egg production. In the present study, in order to understand the molecular mechanisms underlying the dramatic differences of egg number between commercial egg-type chickens and indigenous chickens, we performed a genome-wide association study (GWAS) in a mixed linear model.

**Results:**

We obtained 148 single nucleotide polymorphisms (SNPs) associated with egg number traits (57 significantly, 91 suggestively). Among them, 4 SNPs overlapped with previously reported quantitative trait loci (QTL), including 2 for egg production and 2 for reproductive traits. Furthermore, we identified 32 candidate genes based on the function of the screened genes. These genes were found to be mainly involved in regulating hormones, playing a role in the formation, growth, and development of follicles, and in the development of the reproductive system. Some genes such as *NELL2* (neural EGFL like 2), *KITLG* (KIT ligand), *GHRHR* (Growth hormone releasing hormone receptor), *NCOA1* (Nuclear receptor coactivator 1), *ITPR1* (inositol 1, 4, 5-trisphosphate receptor type 1), *GAMT* (guanidinoacetate N-methyltransferase), and *CAMK4* (calcium/calmodulin-dependent protein kinase IV) deserve our attention and further study since they have been reported to be closely related to egg production, egg number and reproductive traits. In addition, the most significant genomic region obtained in this study was located at 48.61–48.84 Mb on GGA5. In this region, we have repeatedly identified four genes, in which *YY1* (YY1 transcription factor) and *WDR25* (WD repeat domain 25) have been shown to be related to oocytes and reproductive tissues, respectively, which implies that this region may be a candidate region underlying egg number traits.

**Conclusion:**

Our study utilized the genomic information from various chicken breeds or populations differed in the average annual egg number to understand the molecular genetic mechanisms involved in egg number traits. We identified a series of SNPs, candidate genes, or genomic regions that associated with egg number, which could help us in developing the egg production trait in chickens.

**Supplementary Information:**

The online version contains supplementary material available at 10.1186/s12864-021-07755-3.

## Background

Reproduction traits, especially egg production, are the most important economic cares in chickens [1]. Laying performance usually reflects a chicken’s reproductive performance [2]. As an important source of animal protein, the consumption of poultry eggs worldwide has increased significantly over the past few decades [3]. Each person consumes approximately 12.5 kg of eggs per year [4]. Egg consumption may continue to increase with accretion in urban populations [5, 6]. Therefore, it is of great practical and economic significance to understand the genetic mechanisms of chicken reproductive traits. However, egg production is a polygenic genetic trait with low to medium heritability and is affected by both genetic components and environmental factors [7, 8]. It can be evaluated by many indicators such as age at first egg, egg number, egg production rate and so on. The egg number is an important reproductive trait in poultry breeding, an important indicator that can effectively evaluate individual egg production at a certain stage and the fertility of breeding chicken [9].

It is possible to analyze the genetic mechanisms of complex traits by using GWAS with the development of sequencing technology. GWAS can not only take full advantage of molecular markers at the genome level, but, owing to the use of whole genome sequences, avoid the effects of linkage imbalance between SNPs and underlying genes [10]. At present, a few candidate genes and regions related to egg number have been reported based on GWAS technology [9, 11–16]. According to the QTL database [17], 12,782 QTLs related to chicken economic traits have been identified, 332 of which are associated with egg number.

Some commercial lines or populations are intensively selected for their production traits. Rhode Island Red and White Leghorn chickens are well known for their distinguished egg productivity. Dwarf chickens in China have also been developed for egg production. The average annual egg number of them is approximately about 300 eggs. Chinese indigenous chickens grow relatively slowly and are known to produce less than 200 eggs per year. Therefore, we performed a GWAS based on the differences of egg number between egg-type chickens and local chickens to explore the underlying molecular genetic mechanisms and identify candidate genes or genomic regions related to egg number traits. The results of this study are supposed to be beneficial for layer breeding.

## Results

### Population structure testing

**Fig. 1:**
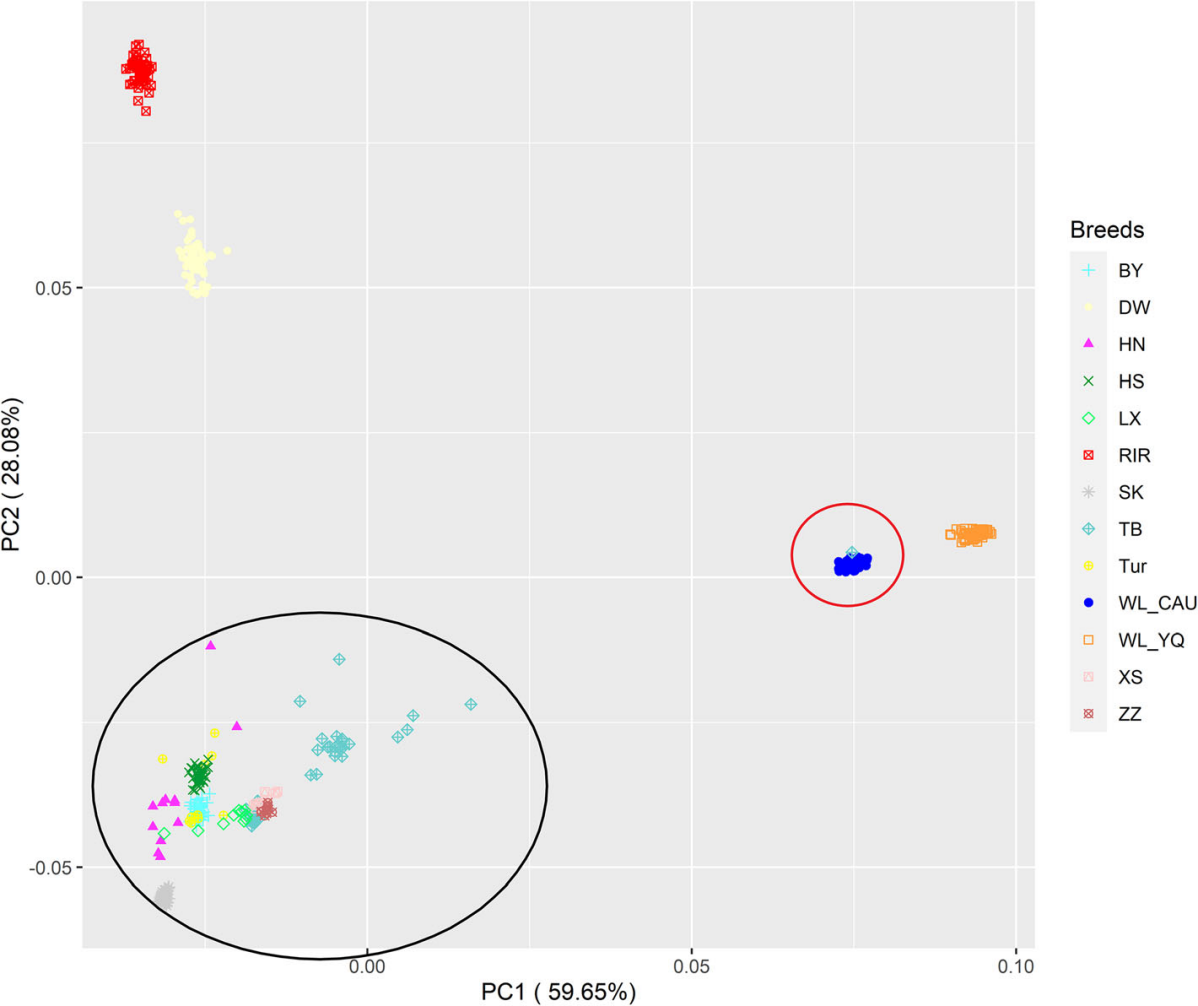
PCA plot of chicken populations in this study. Each color represents a breed and the abbreviations are as defined in Table 3. PC1, principal components one; PC2, principal components two. PC1 and PC2 explain 15.92 and 7.49% of the total variance (or 59.65 and 28.08% of the top three PCs), respectively

Principal component analysis (PCA) using the first two principal components showed that there was an obvious stratification phenomenon between Chinese indigenous chicken breeds (black circle) and commercial egg-type chickens. At the same time, we found a Tibetan chicken was mixed with White Leghorn, as shown by the red circle in Fig. 1 and (see Additional File 4: Figure S1). Meanwhile, WL_CAU and WL_YQ were divided into two groups as they came from two different population. The first principal component (PC1) and the second principal component (PC2) explain 15.92 and 7.49% of the total variance (or 59.65 and 28.08% of the top three PCs), respectively. In GWAS, population stratification might lead to false-positive results. So we used principal components as covariates correct for stratification in this study [18]. When the covariate was added as 1st PC, top two PCs, top three PCs, top four PCs and top five PCs, we performed GWAS and calculated the genomic inflation factors (λ) respectively. λ was 1.004, 0.917, 0.916, 0.891, 0.882, respectively. When the 1st PC was added as a covariate, λ is the closest to 1, indicating that the correction effect of population stratification is the best [19]. So we finally decided to add the 1st PC in the GWAS mixed model to adjust for population stratification.

Admixture software was used to analyze the population structure. We displayed a bar plot based on the cross-validation error rate (Additional file 5: Figure S2). When K = 2, Rhode Island Red and one of the White Leghorn groups (WL_YQ) appeared as two differentiated clusters. When K = 3–4, two White Leghorn populations (WL_CAU, WL_YQ) gathered in the same group. When K = 5, two White Leghorn populations were separated. When K = 6–9, the high productivity layers from four populations (WL_CAU, WL_YQ, RIR, DW) were separated, indicating that the genetic backgrounds of these layers were different (Additional file 4: Figure S1). These results reinforce the subsequent analyses.

### Genome-wide association study

We took the high productivity group as the case and the low productivity group as the control, and performed GWAS on the dramatic differences in egg number between two groups.

**Fig. 2:**
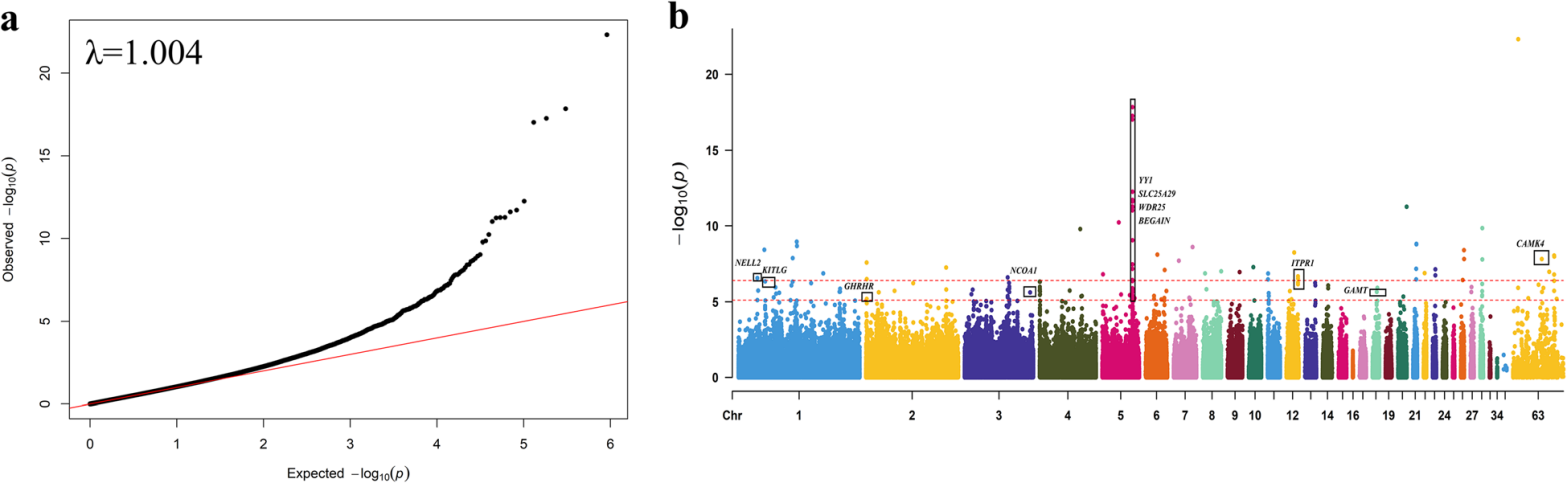
QQ plot and Manhattan of the egg number traits. a The QQ plot shows the expected -log10 *P*-value (the x-axis) plotted against the observed -log10 P-value (y-axis). In the top left of the QQ plot, λ is shown as 1.004. b In Manhattan plot, the x-axis is the position of each SNP on the chicken chromosomes (34, 40, 63 indicate LEG64, W, and Z respectively), and the y-axis is the -log10 P-value. The horizontal red dotted line at the top indicates the genome-wide significant thresholds is 3.91 × 10^− 7^, line at the bottom represents the genome-wide suggestively thresholds is 7.82 × 10^−6^

The QQ plot was presented as Fig. 2a, the λ was equal to 1.004, which means that there was no population stratification phenomenon and the GWAS results were reliable.

After correction, we found 148 SNPs that could be associated with egg number (57 significantly, 91 suggestively) (Additional file 1: Table S1). The global view of *P*-values (in terms of -log10 (P-value)) for all SNPs was represented by a Manhattan plot, as shown in Fig. 2b. Table 1 lists the SNP information that was emphasized in this study. Using Ensembl to annotate related SNPs, we found a total of 68 genes around significant peaks, and identified 32 candidate genes associated with egg number according to their functions (Table 2). Some genes such as *NELL2*, *KITLG*, *GHRHR*, *NCOA1*, *ITPR1*, *GAMT*, and *CAMK4*, which have been proved to be related to egg number, egg production, litter size, or reproductive traits, are worth a deeper exploration [20–33].

**Table 1:** Genome-wide SNPs around significant peaks associated with egg number traits

SNP ID	Chromosome	Posistion^**a**^	***P*** value	Nearest gene
AX-75424481	1	30418287	2.69E-07	***NELL2***
AX-75424489	1	30420285	7.69E-06	***NELL2***
AX-75427140	1	31722672	1.80E-06	***LRIG3***
AX-75448176	1	41739240	3.62E-09	***TSPAN19***
AX-75450814	1	43099910	4.52E-07	***KITLG***
AX-75499001	1	65863432	2.52E-06	***ENSGALG00000046127***
AX-75225234	1	1.18E+08	5.79E-07	***ENSGALG00000036169***
AX-75320912	1	1.63E+08	2.17E-06	***PCDH17***
AX-75322789	1	1.64E+08	2.13E-06	***ENSGALG00000034638***
AX-75322810	1	1.64E+08	1.38E-06	***ENSGALG00000034638***
AX-75965321	2	1068425	2.52E-08	***MINDY4***, ***AQP1***
AX-75993000	2	1215757	6.42E-06	***GHRHR***
AX-75996755	2	1234625	3.13E-07	***GHRHR***
AX-76053674	2	20576504	2.35E-06	***FAM171A1***
AX-80779498	2	46145450	1.88E-06	***TRANK1***
AX-76003273	2	1.3E+08	5.33E-08	***AZIN1***
AX-76003319	2	1.3E+08	1.55E-06	***AZIN1***
AX-76536054	3	69621540	2.42E-07	***NA***
AX-76536071	3	69628923	1.59E-06	***NA***
AX-76536096	3	69655624	4.14E-06	***NA***
AX-76536308	3	69762163	5.02E-06	***NA***
AX-76540844	3	72000979	5.65E-07	***POU3F2***, ***FBXL4***
AX-76541349	3	72227940	9.78E-07	***ENSGALG00000034564***
AX-76541394	3	72243677	7.32E-06	***ENSGALG00000034564***
AX-76541486	3	72303465	2.23E-06	***ENSGALG00000034564***
AX-76541488	3	72304623	1.32E-06	***ENSGALG00000034564***
AX-76401421	3	1.06E+08	2.40E-06	***NCOA1***
AX-76624455	4	141350	1.78E-06	***MSN***
AX-76630762	4	173004	4.71E-07	***MSN***
AX-76645558	4	249014	3.49E-06	***ENSGALG00000044799***
AX-76652327	4	283517	1.22E-06	***HEPH***, ***HSF3***
AX-76658977	4	319076	1.94E-06	***HEPH***, ***GPR83L***
AX-76662643	4	338418	1.02E-06	***GPR83L***, ***ENSGALG00000038728***
AX-80823531	4	394383	1.51E-06	***NA***
AX-76676155	4	408469	4.71E-07	***NA***
AX-76714260	4	612204	3.23E-06	***ENSGALG00000029764***
AX-76718681	4	634832	2.09E-06	***ENSGALG00000029764***
AX-76675026	4	48646624	1.82E-06	***ADGRL3***
AX-76713110	4	67434214	4.37E-06	***ENSGALG00000041624***, ***GABRA2***, ***GABRA4***
AX-76715084	4	68430908	4.09E-06	***GRXCR1***
AX-76776179	5	1133440	1.52E-07	***LUZP2***
AX-76814849	5	30327067	3.26E-06	***RYR3***
AX-76845554	5	44381627	3.63E-06	***CCDC88C***, ***PPP4R3A***
AX-76855266	5	48607009	3.72E-06	***YY1***, ***SLC25A29***
AX-76855305	5	48622650	1.28E-06	***SLC25A29***, ***YY1***
AX-76855335	5	48635048	1.45E-06	***SLC25A29***
AX-76855457	5	48687658	3.62E-07	***WDR25***
AX-76855519	5	48714129	6.08E-06	***WDR25***
AX-76855557	5	48731601	9.56E-12	***WDR25***
AX-76855561	5	48733361	3.33E-08	***WDR25***
AX-76855583	5	48739609	5.29E-12	***BEGAIN***
AX-76855593	5	48745253	5.31E-18	***BEGAIN***
AX-76855630	5	48762022	6.51E-08	***BEGAIN***
AX-76855665	5	48778313	4.22E-07	***BEGAIN***
AX-76855684	5	48786534	9.28E-18	***BEGAIN***
AX-76855699	5	48792877	1.40E-18	***BEGAIN***
AX-76855705	5	48795578	1.94E-12	***BEGAIN***
AX-76855726	5	48804374	2.31E-06	***BEGAIN***
AX-80949259	5	48808664	1.61E-06	***BEGAIN***
AX-76855747	5	48811664	9.02E-10	***BEGAIN***
AX-76855766	5	48820492	5.54E-13	***BEGAIN***
AX-76855815	5	48838402	2.48E-12	***BEGAIN***
AX-76855817	5	48839325	5.76E-12	***BEGAIN***
AX-76900556	6	13775431	4.09E-06	***KCNMA1***
AX-76900629	6	13799359	6.36E-06	***KCNMA1***
AX-76943077	6	30466599	5.62E-06	***NA***
AX-77011457	7	25213170	5.44E-06	***NA***
AX-77014656	7	26516128	6.78E-06	***SLC15A2***, ***IQCB1***
AX-75660603	11	445721	3.26E-07	***CSNK2A2***
AX-75665353	11	607645	1.34E-07	***POLR2C***
AX-75643828	11	1608025	2.68E-06	***HYDIN***
AX-75644090	11	1618412	7.76E-06	***HYDIN***
AX-80948010	11	1754582	4.07E-06	***VAC14***
AX-75703508	12	18557458	5.27E-07	***ITPR1***
AX-75703529	12	18564932	2.08E-07	***ITPR1***
AX-75703541	12	18569966	2.87E-07	***ITPR1***
AX-75703548	12	18572141	6.83E-07	***ITPR1***
AX-75744414	13	16244175	5.66E-07	***VDAC1***
AX-75745363	13	16603463	8.21E-07	***ENSGALG00000029896***
AX-75810149	14	8804646	1.25E-06	***OTOA***
AX-75810223	14	8826708	8.41E-07	***OTOA***
AX-75906835	18	6994110	2.21E-06	***SMURF2***
AX-80787269	18	7876592	1.18E-06	***ARSG***, ***SLC16A6***
AX-76227922	20	8585474	4.52E-06	***ENSGALG00000039201***, ***ENSGALG00000005652***
AX-76249257	21	4983824	3.25E-07	***KAZN***
AX-76249303	21	4996326	6.51E-08	***KAZN***
AX-76250675	21	5263523	1.55E-09	***C1orf158***, ***ENSGALG00000021598***
AX-76288938	23	4139841	1.78E-07	***NA***
AX-80768216	23	4214468	7.13E-08	***AGO1***
AX-76339015	26	3035521	3.58E-07	***NA***
AX-76348623	26	5077655	3.82E-09	***TAF8***
AX-76348772	26	5100952	1.49E-08	***CHIA***
AX-76379082	28	2980442	4.15E-06	***SBNO2***
AX-76379816	28	3170288	3.73E-06	***NA***
AX-76379939	28	3197429	1.98E-06	***GAMT***, ***DAZAP1***
AX-76380005	28	3210016	6.59E-07	***DAZAP1***
AX-76380342	28	3284684	1.57E-08	***SLC39A3***
AX-76380390	28	3295475	1.40E-10	***SLC39A3***, ***DIRAS***
AX-77265370	63	8262232	1.19E-06	***CNTFR***
AX-77206121	63	24853011	5.82E-06	***NA***
AX-77226182	63	46670667	1.48E-08	***CAMK4***
AX-77226564	63	46942411	2.19E-06	***NA***
AX-77228575	63	49223728	1.22E-06	***NA***
AX-77252226	63	66675375	4.08E-07	***MUSK***
AX-77252227	63	66676347	1.56E-07	***MUSK***
AX-77252241	63	66683942	1.89E-06	***MUSK***
AX-77252265	63	66694071	8.69E-09	***MUSK***
AX-80869954	63	66732038	1.01E-08	***MUSK***

^a^ Physical position

**Table 2:** Details for 32 candidate genes that influence egg number traits in different ways

Ways impacting reproductive traits	Genes
Regulating hormone level	***NELL2***, ***AQP1***, ***AZIN1***, ***POU3F2***, ***POLR2C***, ***GPR83L***, ***ENSGALG00000038728***, ***RYR3***, ***ITPR1***, ***CNTFR***
Influencing the formation, growth, development of follicle	***LRIG3***, ***KITLG***, ***PCDH17***, ***GHRHR***, ***ADGRL3***, ***CCDC88C***, ***YY1***, ***CSNK2A2***, ***POLR2C***, ***ITPR1***, ***SMURF2***, ***AGO1***, ***CHIA***, ***DAZAP1***, ***DIRAS1***, ***MUSK***, ***CAMK4***
Influencing the development of the reproductive system	***WDR25***, ***ENSGALG00000029896***, ***ENSGALG00000039201***, ***GAMT***, ***DAZAP1***, ***ENSGALG00000005652***, ***SLC39A3***, ***HYDIN***

In addition, the most significant peak in this study was located at 48.61–48.84 Mb on chromosome 5. The chi-square test was carried out to compare the allele frequencies of the significant SNPs identified in this region between the high and low productivity groups. The results showed that the allele frequencies of these 20 SNPs were significantly different between the two groups (Additional file 2: Table S2). At the same time, four genes were repeatedly identified in this region. *YY1* is involved in oocyte growth and maturation [34]. A member of the solute carrier family 25 (*SLC25A29*) is involved in the transport of amino acids. *WDR25* may be related to the reproductive tissues [35]. Brain enriched guanylate kinase (*BEGAIN*) is a gene specifically expressed in the brain and is involved in the regulation of postsynaptic neurotransmitter receptor activity [36].

### Comparing with previously reported QTLs

Through Animal QTLdb, we detected 4 QTLs that overlapped with SNPs obtained from this study. Two of these 4 QTLs were associated with egg production, including 1 with egg production rate and 1 with small yellow follicle number. The remaining QTLs were related to reproductive traits, including 2 with ovary weight (Additional file 3: Table S3).

## Discussion

### GWAS and QTL overlapping

An important condition for GWAS to achieve better results is to eliminate false associations caused by differences in allele frequencies arising from population stratification, recessive kinship, and genotyping errors [37]. The GEMMA adopted in this study considers the group stratification and sample structure. At the same time, we also added the PC1 as a covariate to reduce the group stratification effect. The results of the QQ plot and λ show that the correction effect is good, and there is no population stratification phenomenon.

Four of the 148 QTLs in this study were those identified in previous studies. Among these overlapping QTLs, 2 QTLs were associated with egg production. This reinforces the results of our study. AX-75745363 is located the *ENSGALG00000029896* gene on chromosome 13. AX-76715084 is located 0.046 Mb upstream of the *GRXCR1* (glutaredoxin and cysteine-rich domain containing 1) gene on chromosome 4. Although these genes have not been very well studied in chickens, and their functions have not been fully elucidated, they provide a reference and idea to understand the molecular mechanism for egg number traits.

### Candidate genes

As far as we know, this study has the largest variety of breeds so far in the research of reproductive traits in chicken, which not only improves the detection ability of related QTLs, but also allows us to detect some QTLs related to fat and heat resistance. We speculate that this was due to the relatively slow fat formation and deposition [38, 39] and the relatively high heat generation of layers [40]. Thus, we identified 32 candidate genes based on their function. These genes mainly affect egg number in three ways (Table 2). Some genes regulate hormone levels, including gonadotropin-releasing hormone (GNRH), oxytocin (OXT), growth hormone (GH), and thyroid hormone (TH). All these hormones play a vital role in the female reproductive system [41–46]. Some genes affect egg number traits by affecting the growth and development of follicles. It is well known that the growth and development of follicles are critical for reproductive function, especially in chickens. The remaining genes directly affect reproductive system development. Among these 32 candidate genes, we also found 7 important genes that have been identified as related to reproductive traits such as egg production, egg number and litter size in previous studies, which further validates our findings. *NELL2* and *KITLG* are located on GGA1. *NELL2* not only affects the synthesis and secretion of GNRH [47], but has also been shown to be involved in maintaining the normal female reproductive cycle of mammals [20]. *KITLG* plays an important role in the growth and development of follicles [21, 48]. It has been shown to be related to the litter size of goat and sheep, and has been considered an excellent candidate gene for reproductive traits of humans and livestock [21–23]. Therefore, it is reasonable to speculate that *KITLG* has an important impact on egg number traits. *GHRHR* located on GGA2 participates in the secretion and synthesis of GH. It is believed to be involved in the growth and reproduction of livestock [49]. Liu et al. identified three SNPs in the *GHRHR* promoter that are significantly related to egg number traits in Beijing You chickens [24]. *NCOA1*, located on GGA3, is involved in regulating signal pathways mediated by TH and estrogen. It has not only been shown to be an important gene that influences reproductive traits in pigs and sheep, but also related to egg production, fertility, and reproductive traits in chicken [25–30]. *ITPR1* repeatedly identified in the 18.56–18.57 Mb on chromosome 12 can not only participate in the signaling pathway of GnRH, estrogen, and the synthesis and secretion of GH and TH, but also affects the growth and differentiation of follicles. In addition, *ITPR1* has been reported to be involved in the transport of Ca^2+^ and may be associated with egg number [31]. This suggests that this region located on chromosome 12 and the *ITPR1* gene may be important for chicken egg number traits. *GAMT* located on GGA28 has been shown to be associated with the reproductive system and development [32]. *CAMK4* located on chromosome Z is involved in the signaling pathway of OXT and may play a role in the development of follicles and ovulation [50]. It is believed to play a significant role in the reproductive processes of females [33].

However, *NELL2*, *GAMT*, and *CAMK4* have not been studied before in chickens, and the results of this study may pave the way for future researchers to explore the relationship between these genes and egg number traits. Also, the specific functions of these genes need to be further verified.

### Candidate region

In this study, the most significant peak obtained was located at 48.61–48.84 Mb region on GGA5. In this region, *YY1*, *SLC25A29*, *WDR25*, and *BEGAIN* were annotated. Among them, *YY1* and *WDR25* have been shown to be related to oocytes and reproductive tissues, respectively [34, 35]. However, there is no concrete literature to prove that they are associated with egg number traits in chickens, thus further research is still required. At the same time, interestingly enough, a number of studies have detected regions associated with egg number traits on chromosome 5 [12, 15]. The region identified in our study was about 1.2 Mb away from the QTL reported by Zhang et al. [15]. Although the results are different, it has once again proved that chromosome 5 is an important candidate region that affects the reproductive traits of chickens.

## Conclusions

In this study, we performed a GWAS based on the difference of egg number between high productivity layers and Chinese indigenous chickens and identified a series of SNPs and candidate genes related to reproductive traits. Four of the SNP effects overlapped with previously reported QTL regions, which supports the results of this study. These results may help us to better understand the molecular mechanisms underlying reproductive traits in chickens and even other species.

## Materials and methods

### Experimental animals

For this study, 442 chicks were available. Among them, White Leghorn and Rhode Island Red are intensively selected commercial breed, Dwarf Chicken is a synthetic layer line. Both they are egg-type chickens and produce about 300 eggs per year. They were placed in the high egg productivity group in this study. The other ten Chinese indigenous breeds laying less than 200 eggs annually were classified into the low egg productivity group. The details of the samples are presented in Table 3.

**Table 3:** Summary of phenotypic data

Group	Breeds	Abbreviation	Sample size	Average annual egg number^n^	Total^b^
High productivity	White Leghorn^a^	WL	100	315	220
	Dwarf^c^	DW	60	280	
	Rhode Island Red^d^	RIR	60	310	
low productivity	Beijing You^e^	BY	42	110	222
	Henan Game^f^	HN	13	100	
	Xishuangbanna Game^g^	XS	10	110	
	Turpan^h^	TU	11	70	
	Zhangzhou Game^i^	ZZ	10	80	
	Luxi Game^j^	LX	10	40–60	
	Tibetan^k^	TB	42	40–80	
	Hongshan^l^	HS	42	116	
	Taihe Silkies^m^	SK	42	110	
Total	–	–	–	–	442

^a^White Leghorn came from two different groups. Among them, 40 samples obtained from the Experimental Chicken Farm at the China Agricultural University, 60 samples obtained from Beijing Yanqing Commercial Layer Breeding Company. Their abbreviations are WL_CAU and WL_YQ, respectively

^b^Regarding the sample size, we tried to make the ratio between case and control was 1:1 to avoid the large number difference between the two groups interfering the results

^c^Dwarf chickens obtained from Beijing Yanqing Commercial Layer Breeding Company

^d^Rhode Island Red obtained from Beijing

^e^Beijing You chickens obtained from Beijing

^f^Henan Game obtained from Henan province

^g^Xishuangbanna Game obtained from Xishuangbanna, Yunnan province

^h^Turpan obtained from Turpan, Xinjiang

^i^Zhangzhou Game obtained from Fujiang province

^j^Luxi Game obtained from Shandong province

^k^Tibetan obtained from four different places, including the Experimental Chicken Farm at the China Agricultural University, Nimu, Tibet, Naidong, Tibet and Lingzhi, Tibet

^l^Hongshan chickens obtained from Hubei province

^m^Taihe Silkies chickens obtained from the Experimental Chicken Farm at the China Agricultural University

^n^Refer to the poultry genetic resources in China

### Genotyping and quality control

In total, blood samples from 442 chickens from the high and low egg productivity groups were collected by standard venipuncture. After DNA extraction using the standard phenol/chloroform method [51], the chickens were genotyped using a 600 K Affymetrix Axiom Chicken Genotyping Array with a total of 580,961 SNPs [52]. Quality control was performed using Plink v1.9 [53]. SNPs with a minor allele frequency ≥ 1% and genotyping rate ≥ 98% were retained. Individuals with a genotype deletion rate of > 5% were excluded. SNPs with Hardy-Weinberg equilibrium *P* < 10^− 6^ were eliminated. After filtering, 439 chickens, including 218 in high and 221 in low egg productivity groups, and 456,647 SNPs were retained for further analyses.

### Population structure analysis

Prior to GWAS, population structure was examined by PCA. Plink 1.9 was used to determine the population structure and generate eigenvectors and eigenvalues, and the “ggplot2” package in R studio was used to visualize the results of PCA. We selected the first two principal components with the largest variance interpretation rate as the horizontal and vertical coordinates to create a PCA plot. At the same time, we calculated the principal component contribution rate based on 439 eigenvalues.

We retained relatively dependent SNPs with the plink ‘--indep-pairwise 25 5 0.2’ command. The genetic structure was estimated using Admixture software [54]. We calculated the ancestor coefficient matrix, simulated the situation of genetic clusters (K) from 1 to 20, and computed the cross-validation error rate. Furthermore, we used an online pophelper to display a population structure bar plot (http://pophelper.com/) [55].

### Genome-wide association study

GWAS analyses of the egg number were performed using a univariate mixed linear model in GEMMA [56]. In the current study, only the PC1 was used as a covariate to correct population stratification. The model is as follows:

$$ \mathrm{y}=\mathrm{W}\upalpha +\mathrm{x}\upbeta +\mathrm{u}+\upvarepsilon $$

where *y* denotes a phenotypic value vector of 439 individuals, *W* is a matrix of covariates (fixed effects that contain a column of 1 s and the first principal component), *α* represents a vector of the corresponding coefficients consisting of intercepts, *x* is a vector of marker genotypes, *β* is the effect size of a marker, *u* is a vector of random effects with a covariance structure that follows a normal distribution as *u* ~ N (0, KVg), where K is a genetic relationship matrix and Vg is the polygenic additive variance; and *ɛ* is a vector of random residuals. In this study, the Wald statistic was used to test each SNP.

Manhattan and Quantile-Quantile (QQ) plot were made by R package “CMplot” and “qqman” respectively, and we also calculated λ based on *p*-values from GWAS to judge the degree of false-positive [57]. λ was calculated by the median of the resulting chi-squared test statistics divided by the expected median of the chi-squared distribution. We set the median of a chi-squared distribution with one degree of freedom was 0.454 in this study.

The traditional Bonferroni correction is too strict, resulting in a higher false-negative rate and omission of some SNPs truly associated with the target trait [58]. Therefore, in this study, we calculated the sum of the number of independent SNPs and LD blocks for correction [59]. The effective number of independent tests was 127,862 in this study. Hence, the threshold *P* value was adjusted to 3.91 × 10^− 7^ for a genome-wide significance level, and 7.82 × 10^− 6^ for a genome-wide suggestive significance level. This means that SNPs with *P* values below 7.82 × 10^− 6^ are considered and may be associated with egg number traits.

### Bioinformatics analysis of candidate genes

We identified candidate genes by searching for the nearest genes located within 400 bp upstream or downstream of the significant associated SNPs and annotated based on the Galgal 5.0 assembly supported by Ensembl (http://www.ensembl.org/index.html) databases. We then checked the biological functions of these genes in PubMed (https://pubmed.ncbi.nlm.nih.gov).

### Overlap with known QTLs

In addition, regions within 100 kb of a candidate SNP were searched for previously reported QTLs with egg number, egg production or reproductive traits in the chicken QTL database (https://www.animalgenome.org/cgi-bin/QTLdb/GG/index).

## Supplementary Information

**Additional file 1: Table S1.** Genome-wide SNPs associated with egg number traits.

**Additional file 2: Table S2**. Difference of the allele frequencies of the significant SNPs located at 48.61–48.84 Mb on chromosome 5 between the high and low productivity groups.

**Additional file 3: Table S3.** Results for comparison with previously reported QTLs. ^a^ The unit is Mb. U and D represent that SNP located upstream and downstream of the gene, respectively. ^b^ The unit is Mb. U and D represent that SNP located upstream and downstream of the gene, respectively.

**Additional file 4: Figure S1**. Admixture plot. Each color represents separate groups, each line represents a group value.

**Additional file 5: Figure S2**. The line chart of cross validation error. The coefficient of variation value for each K-value, the accessions were divided into 20 subgroups (there was minimum K-value when K = 12).

## Data Availability

The datasets generated for this study can be found in FigShare https://figshare.com/s/7592d6524d1af7acfdb7.

## Abbreviations

GWAS: Genome-wide association study
SNPs: Single nucleotide polymorphisms
QTL: Quantitative trait loci
PCA: Principal component analysis
λ: Genomic inflation factors
