# Supplementary material for: Identification of candidate genomic regions for chicken egg number traits based on genome-wide association study
Source: BMC Genomics. 2021 Aug 10;22:610. doi: 10.1186/s12864-021-07755-3 (PMC8356427; doi:10.1186/s12864-021-07755-3)
Supplement: Supplementary file 1 — Additional file 1: Table S1. Genome-wide SNPs associated with egg number traits. [file 12864_2021_7755_MOESM1_ESM.docx]

| Table S1 Genome-wide SNPs associated with egg number traits. | | | | |
| --- | --- | --- | --- | --- |
| **SNP ID** | **Chrosome** | **Posistion** | **P vaule** | **Nearest gene** |
| AX-76288641 | 0 | 0 | 2.01E-06 | ***NA*** |
| AX-75424481 | 1 | 30418287 | 2.69E-07 | ***NELL2*** |
| AX-75424489 | 1 | 30420285 | 7.69E-06 | ***NELL2*** |
| AX-75427140 | 1 | 31722672 | 1.80E-06 | ***LRIG3*** |
| AX-75448176 | 1 | 41739240 | 3.62E-09 | ***TSPAN19*** |
| AX-75450814 | 1 | 43099910 | 4.52E-07 | ***KITLG*** |
| AX-75480153 | 1 | 56943004 | 5.04E-06 | ***DENND2A*** |
| AX-75486162 | 1 | 59769134 | 1.10E-06 | ***TMTC1*** |
| AX-75488430 | 1 | 60860595 | 2.91E-06 | ***ERC1*** |
| AX-75499001 | 1 | 65863432 | 2.52E-06 | ***ENSGALG00000046127*** |
| AX-75534946 | 1 | 84258369 | 7.80E-06 | ***COL8A1*** |
| AX-80890167 | 1 | 85607083 | 8.19E-07 | ***ZPLD1*** |
| AX-75541864 | 1 | 87582860 | 4.73E-07 | ***KIAA1524*** |
| AX-75541873 | 1 | 87587117 | 4.73E-07 | ***KIAA1524*** |
| AX-75541963 | 1 | 87635530 | 1.30E-08 | ***SH2D1B*** |
| AX-75555852 | 1 | 94282354 | 1.12E-09 | ***NA*** |
| AX-75556507 | 1 | 94635065 | 2.03E-09 | ***NA*** |
| AX-75225234 | 1 | 1.18E+08 | 5.79E-07 | ***ENSGALG00000036169*** |
| AX-75267293 | 1 | 1.37E+08 | 1.31E-07 | ***TMEM255B*** |
| AX-75320912 | 1 | 1.63E+08 | 2.17E-06 | ***PCDH17*** |
| AX-75322789 | 1 | 1.64E+08 | 2.13E-06 | ***ENSGALG00000034638*** |
| AX-75322810 | 1 | 1.64E+08 | 1.38E-06 | ***ENSGALG00000034638*** |
| AX-75965321 | 2 | 1068425 | 2.52E-08 | ***MINDY4***, ***AQP*** |
| AX-75993000 | 2 | 1215757 | 6.42E-06 | ***GHRHR*** |
| AX-75996755 | 2 | 1234625 | 3.13E-07 | ***GHRHR*** |
| AX-76053674 | 2 | 20576504 | 2.35E-06 | ***FAM171A1*** |
| AX-80779498 | 2 | 46145450 | 1.88E-06 | ***TRANK1*** |
| AX-76152312 | 2 | 76468530 | 5.85E-07 | ***ANKH*** |
| AX-76003273 | 2 | 1.3E+08 | 5.33E-08 | ***AZIN1*** |
| AX-76003319 | 2 | 1.3E+08 | 1.55E-06 | ***AZIN1*** |
| AX-76410884 | 3 | 10971427 | 3.12E-06 | ***ETAA1*** |
| AX-76417317 | 3 | 13012723 | 1.59E-06 | ***NA*** |
| AX-76536054 | 3 | 69621540 | 2.42E-07 | ***NA*** |
| AX-76536071 | 3 | 69628923 | 1.59E-06 | ***NA*** |
| AX-76536096 | 3 | 69655624 | 4.14E-06 | ***NA*** |
| AX-76536308 | 3 | 69762163 | 5.02E-06 | ***NA*** |
| AX-76540844 | 3 | 72000979 | 5.65E-07 | ***POU3F2***, ***FBXL4*** |
| AX-76541349 | 3 | 72227940 | 9.78E-07 | ***ENSGALG00000034564*** |
| AX-76541394 | 3 | 72243677 | 7.32E-06 | ***ENSGALG00000034564*** |
| AX-76541486 | 3 | 72303465 | 2.23E-06 | ***ENSGALG00000034564*** |
| AX-76541488 | 3 | 72304623 | 1.32E-06 | ***ENSGALG00000034564*** |
| AX-76401421 | 3 | 1.06E+08 | 2.40E-06 | ***NCOA1*** |
| AX-76624455 | 4 | 141350 | 1.78E-06 | ***MSN*** |
| AX-76630762 | 4 | 173004 | 4.71E-07 | ***MSN*** |
| AX-76645558 | 4 | 249014 | 3.49E-06 | ***ENSGALG00000044799*** |
| AX-76652327 | 4 | 283517 | 1.22E-06 | ***HEPH***, ***HSF3*** |
| AX-76658977 | 4 | 319076 | 1.94E-06 | ***HEPH***, ***GPR83L*** |
| AX-76662643 | 4 | 338418 | 1.02E-06 | ***GPR83L***, ***ENSGALG00000038728*** |
| AX-80823531 | 4 | 394383 | 1.51E-06 | ***NA*** |
| AX-76676155 | 4 | 408469 | 4.71E-07 | ***NA*** |
| AX-76714260 | 4 | 612204 | 3.23E-06 | ***ENSGALG00000029764*** |
| AX-76718681 | 4 | 634832 | 2.09E-06 | ***ENSGALG00000029764*** |
| AX-76675026 | 4 | 48646624 | 1.82E-06 | ***ADGRL3*** |
| AX-76709631 | 4 | 65674981 | 1.64E-10 | ***KIT*** |
| AX-76713110 | 4 | 67434214 | 4.37E-06 | ***ENSGALG00000041624***, ***GABRA2***, ***GABRA4*** |
| AX-76715084 | 4 | 68430908 | 4.09E-06 | ***GRXCR1*** |
| AX-76776179 | 5 | 1133440 | 1.52E-07 | ***LUZP2*** |
| AX-76806561 | 5 | 26655630 | 5.87E-11 | ***ENSGALG00000033973*** |
| AX-76814849 | 5 | 30327067 | 3.26E-06 | ***RYR3*** |
| AX-76845554 | 5 | 44381627 | 3.63E-06 | ***CCDC88C***, ***PPP4R3A*** |
| AX-76855266 | 5 | 48607009 | 3.72E-06 | ***YY1*** |
| AX-76855305 | 5 | 48622650 | 1.28E-06 | ***SLC25A29***, ***YY1*** |
| AX-76855335 | 5 | 48635048 | 1.45E-06 | ***SLC25A29*** |
| AX-76855457 | 5 | 48687658 | 3.62E-07 | ***WDR25*** |
| AX-76855519 | 5 | 48714129 | 6.08E-06 | ***WDR25*** |
| AX-76855557 | 5 | 48731601 | 9.56E-12 | ***WDR25*** |
| AX-76855561 | 5 | 48733361 | 3.33E-08 | ***WDR25*** |
| AX-76855583 | 5 | 48739609 | 5.29E-12 | ***BEGAIN*** |
| AX-76855593 | 5 | 48745253 | 5.31E-18 | ***BEGAIN*** |
| AX-76855630 | 5 | 48762022 | 6.51E-08 | ***BEGAIN*** |
| AX-76855665 | 5 | 48778313 | 4.22E-07 | ***BEGAIN*** |
| AX-76855684 | 5 | 48786534 | 9.28E-18 | ***BEGAIN*** |
| AX-76855699 | 5 | 48792877 | 1.40E-18 | ***BEGAIN*** |
| AX-76855705 | 5 | 48795578 | 1.94E-12 | ***BEGAIN*** |
| AX-76855726 | 5 | 48804374 | 2.31E-06 | ***BEGAIN*** |
| AX-80949259 | 5 | 48808664 | 1.61E-06 | ***BEGAIN*** |
| AX-76855747 | 5 | 48811664 | 9.02E-10 | ***BEGAIN*** |
| AX-76855766 | 5 | 48820492 | 5.54E-13 | ***BEGAIN*** |
| AX-76855815 | 5 | 48838402 | 2.48E-12 | ***BEGAIN*** |
| AX-76855817 | 5 | 48839325 | 5.76E-12 | ***BEGAIN*** |
| AX-76864957 | 5 | 52414840 | 5.68E-06 | ***NA*** |
| AX-76900556 | 6 | 13775431 | 4.09E-06 | ***KCNMA1*** |
| AX-76900629 | 6 | 13799359 | 6.36E-06 | ***KCNMA1*** |
| AX-76914014 | 6 | 19118336 | 7.55E-09 | ***TMEM72*** |
| AX-76930453 | 6 | 25657784 | 6.94E-06 | ***NA*** |
| AX-76943077 | 6 | 30466599 | 5.62E-06 | ***NA*** |
| AX-76944573 | 6 | 31031659 | 7.79E-08 | ***ENSGALG00000033285*** |
| AX-76952045 | 6 | 33728812 | 1.90E-06 | ***NA*** |
| AX-80860981 | 7 | 8304966 | 1.93E-08 | ***TMEFF2*** |
| AX-77011457 | 7 | 25213170 | 5.44E-06 | ***NA*** |
| AX-77014656 | 7 | 26516128 | 6.78E-06 | ***SLC15A2***, ***IQCB1*** |
| AX-77025318 | 7 | 30491355 | 2.37E-09 | ***TMEM163*** |
| AX-77108458 | 8 | 3617571 | 1.32E-07 | ***RGS1***, ***RGS2***, ***RGS13*** |
| AX-77113953 | 8 | 5896668 | 1.48E-06 | ***XPR1*** |
| AX-77106132 | 8 | 29777765 | 9.50E-08 | ***LHX8***, ***TYW3*** |
| AX-77150939 | 9 | 19409166 | 1.09E-07 | ***FNDC3B*** |
| AX-75619037 | 10 | 7302581 | 5.05E-08 | ***TCF12***, ***CGNL1*** |
| AX-75660603 | 11 | 445721 | 3.26E-07 | ***CSNK2A2*** |
| AX-75665353 | 11 | 607645 | 1.34E-07 | ***POLR2C*** |
| AX-75643828 | 11 | 1608025 | 2.68E-06 | ***HYDIN*** |
| AX-75644090 | 11 | 1618412 | 7.76E-06 | ***HYDIN*** |
| AX-80948010 | 11 | 1754582 | 4.07E-06 | ***VAC14*** |
| AX-75716772 | 12 | 5402523 | 1.95E-06 | ***IQSEC1*** |
| AX-75724990 | 12 | 8188245 | 6.78E-06 | ***ERC2*** |
| AX-75685213 | 12 | 12455744 | 5.52E-09 | ***NA*** |
| AX-75703508 | 12 | 18557458 | 5.27E-07 | ***ITPR1*** |
| AX-75703529 | 12 | 18564932 | 2.08E-07 | ***ITPR1*** |
| AX-75703541 | 12 | 18569966 | 2.87E-07 | ***ITPR1*** |
| AX-75703548 | 12 | 18572141 | 6.83E-07 | ***ITPR1*** |
| AX-75744414 | 13 | 16244175 | 5.66E-07 | ***VDAC1*** |
| AX-75745363 | 13 | 16603463 | 8.21E-07 | ***ENSGALG00000029896*** |
| AX-75810149 | 14 | 8804646 | 1.25E-06 | ***OTOA*** |
| AX-75810223 | 14 | 8826708 | 8.41E-07 | ***OTOA*** |
| AX-75906835 | 18 | 6994110 | 2.21E-06 | ***SMURF2*** |
| AX-80787269 | 18 | 7876592 | 1.18E-06 | ***SLC16A6*** |
| AX-76227922 | 20 | 8585474 | 4.52E-06 | ***ENSGALG00000039201***, ***ENSGALG00000005652*** |
| AX-80872656 | 20 | 13885974 | 5.46E-12 | ***NA*** |
| AX-76249257 | 21 | 4983824 | 3.25E-07 | ***KAZN*** |
| AX-76249303 | 21 | 4996326 | 6.51E-08 | ***KAZN*** |
| AX-76250675 | 21 | 5263523 | 1.55E-09 | ***C1orf158***, ***ENSGALG00000021598*** |
| AX-76261071 | 22 | 1636463 | 1.27E-07 | ***DUSP26***, ***TTI2***, ***RNF122*** |
| AX-76288938 | 23 | 4139841 | 1.78E-07 | ***NA*** |
| AX-80768216 | 23 | 4214468 | 7.13E-08 | ***AGO1*** |
| AX-76339015 | 26 | 3035521 | 3.58E-07 | ***NA*** |
| AX-76348623 | 26 | 5077655 | 3.82E-09 | ***TAF8*** |
| AX-76348772 | 26 | 5100952 | 1.49E-08 | ***CHIA*** |
| AX-76351954 | 27 | 1296472 | 2.27E-06 | ***WNT3*** |
| AX-76354045 | 27 | 1789630 | 1.06E-06 | ***GH***, ***RDM1*** |
| AX-76379082 | 28 | 2980442 | 4.15E-06 | ***SBNO2*** |
| AX-76379816 | 28 | 3170288 | 3.73E-06 | ***NA*** |
| AX-76379939 | 28 | 3197429 | 1.98E-06 | ***GAMT***, ***DAZAP1*** |
| AX-76380005 | 28 | 3210016 | 6.59E-07 | ***DAZAP1*** |
| AX-76380342 | 28 | 3284684 | 1.57E-08 | ***SLC39A3*** |
| AX-76380390 | 28 | 3295475 | 1.40E-10 | ***SLC39A3***, ***DIRAS*** |
| AX-77265370 | 63 | 8262232 | 1.19E-06 | ***CNTFR*** |
| AX-77269564 | 63 | 8523916 | 4.76E-23 | ***DNAJB5*** |
| AX-77206121 | 63 | 24853011 | 5.82E-06 | ***NA*** |
| AX-77221928 | 63 | 41389657 | 7.46E-07 | ***DAPK1*** |
| AX-77226182 | 63 | 46670667 | 1.48E-08 | ***CAMK4*** |
| AX-77226564 | 63 | 46942411 | 2.19E-06 | ***NA*** |
| AX-77228575 | 63 | 49223728 | 1.22E-06 | ***NA*** |
| AX-77241427 | 63 | 58611960 | 1.03E-07 | ***MUSK*** |
| AX-77252226 | 63 | 66675375 | 4.08E-07 | ***MUSK*** |
| AX-77252227 | 63 | 66676347 | 1.56E-07 | ***MUSK*** |
| AX-77252241 | 63 | 66683942 | 1.89E-06 | ***MUSK*** |
| AX-77252265 | 63 | 66694071 | 8.69E-09 | ***MUSK*** |
| AX-80869954 | 63 | 66732038 | 1.01E-08 | ***MUSK*** |
| AX-77258647 | 63 | 70799618 | 6.48E-06 | ***TMOD1*** |
