# Supplementary material for: Identification of candidate genomic regions for chicken egg number traits based on genome-wide association study
Source: BMC Genomics. 2021 Aug 10;22:610. doi: 10.1186/s12864-021-07755-3 (PMC8356427; doi:10.1186/s12864-021-07755-3)
Supplement: Supplementary file 2 — Additional file 2: Table S2. Difference of the allele frequencies of the significant SNPs located at 48.61–48.84 Mb on chromosome 5 between the high and low productivity groups. [file 12864_2021_7755_MOESM2_ESM.docx]

| Table S2 Difference of the allele frequencies of the significant SNPs located at 48.61-48.84Mb on chromosome 5 between the high and low productivity groups. | | | | | | | | | | | | | |
| --- | --- | --- | --- | --- | --- | --- | --- | --- | --- | --- | --- | --- | --- |
| **SNP** | **Group** | **Alle frequency** | | **χ^2^(p-vaule)** |  |  |  |  |  |  |  |  |  |
|  |  | **C** | **T** |  |  |  |  |  |  |  |  |  |  |
| AX-76855266 | High | 0.32569 | 0.67431 | 147.699(<0.01) |  |  |  |  |  |  |  |  |  |
|  | Low | 0.89367 | 0.10634 |  |  |  |  |  |  |  |  |  |  |
| AX-76855305 | High | 0.46544 | 0.53456 | 114.715(<0.01) |  |  |  |  |  |  |  |  |  |
|  | Low | 0.93213 | 0.06787 |  |  |  |  |  |  |  |  |  |  |
| AX-76855335 | High | 0.48853 | 0.51147 | 103.499(<0.01) |  |  |  |  |  |  |  |  |  |
|  | Low | 0.92694 | 0.07306 |  |  |  |  |  |  |  |  |  |  |
| AX-76855557 | High | 0.5576 | 0.4424 | 129.256(<0.01) |  |  |  |  |  |  |  |  |  |
|  | Low | 0.05708 | 0.94292 |  |  |  |  |  |  |  |  |  |  |
| AX-76855561 | High | 0.47857 | 0.52143 | 126.531(<0.01) |  |  |  |  |  |  |  |  |  |
|  | Low | 0.95701 | 0.04299 |  |  |  |  |  |  |  |  |  |  |
| AX-76855665 | High | 0.46101 | 0.53899 | 96.619(<0.01) |  |  |  |  |  |  |  |  |  |
|  | Low | 0.89819 | 0.10181 |  |  |  |  |  |  |  |  |  |  |
| AX-76855684 | High | 0.54128 | 0.45872 | 130.728(<0.01) |  |  |  |  |  |  |  |  |  |
|  | Low | 0.04525 | 0.95475 |  |  |  |  |  |  |  |  |  |  |
| AX-76855699 | High | 0.54128 | 0.45872 | 130.728(<0.01) |  |  |  |  |  |  |  |  |  |
|  | Low | 0.04751 | 0.95249 |  |  |  |  |  |  |  |  |  |  |
| AX-76855705 | High | 0.53687 | 0.46313 | 131.921(<0.01) |  |  |  |  |  |  |  |  |  |
|  | Low | 0.04299 | 0.95701 |  |  |  |  |  |  |  |  |  |  |
| AX-76855766 | High | 0.45872 | 0.54128 | 111.127(<0.01) |  |  |  |  |  |  |  |  |  |
|  | Low | 0.92431 | 0.07569 |  |  |  |  |  |  |  |  |  |  |
| AX-76855815 | High | 0.54378 | 0.45622 | 105.175(<0.01) |  |  |  |  |  |  |  |  |  |
|  | Low | 0.08986 | 0.91014 |  |  |  |  |  |  |  |  |  |  |
| **SNP** | **Group** | **Alle frequency** | | **χ^2^(p-vaule)** |  |  |  |  |  |  |  |  |  |
|  |  | **A** | **G** |  |  |  |  |  |  |  |  |  |  |
| AX-76855457 | High | 0.50691 | 0.49309 | 91.886(<0.01) |  |  |  |  |  |  |  |  |  |
|  | Low | 0.0905 | 0.9095 |  |  |  |  |  |  |  |  |  |  |
| AX-76855519 | High | 0.52294 | 0.47706 | 78.738(<0.01) |  |  |  |  |  |  |  |  |  |
|  | Low | 0.125 | 0.875 |  |  |  |  |  |  |  |  |  |  |
| AX-76855593 | High | 0.54128 | 0.45872 | 127.77(<0.01) |  |  |  |  |  |  |  |  |  |
|  | Low | 0.04977 | 0.95023 |  |  |  |  |  |  |  |  |  |  |
| AX-76855726 | High | 0.54128 | 0.45872 | 82.854(<0.01) |  |  |  |  |  |  |  |  |  |
|  | Low | 0.13122 | 0.86878 |  |  |  |  |  |  |  |  |  |  |
| AX-76855747 | High | 0.45872 | 0.54128 | 108.527(<0.01) |  |  |  |  |  |  |  |  |  |
|  | Low | 0.91855 | 0.08145 |  |  |  |  |  |  |  |  |  |  |
| AX-76855817 | High | 0.54608 | 0.45392 | 75.681(<0.01) |  |  |  |  |  |  |  |  |  |
|  | Low | 0.08409 | 0.91591 |  |  |  |  |  |  |  |  |  |  |
| **SNP** | **Group** | **Alle frequency** | | **χ^2^(p-vaule)** |  |  |  |  |  |  |  |  |  |
|  |  | **A** | **C** |  |  |  |  |  |  |  |  |  |  |
| AX-76855583 | High | 0.44495 | 0.55505 | 130.31(<0.01) |  |  |  |  |  |  |  |  |  |
|  | Low | 0.9457 | 0.0543 |  |  |  |  |  |  |  |  |  |  |
| AX-76855630 | High | 0.45872 | 0.54128 | 87.148(<0.01) |  |  |  |  |  |  |  |  |  |
|  | Low | 0.87955 | 0.12046 |  |  |  |  |  |  |  |  |  |  |
| **SNP** | **Group** | **Alle frequency** | | **χ^2^(p-vaule)** |  |  |  |  |  |  |  |  |  |
|  |  | **A** | **T** |  |  |  |  |  |  |  |  |  |  |
| AX-80949259 | High | 0.44419 | 0.55581 | 106.128(<0.01) |  |  |  |  |  |  |  |  |  |
|  | Low | 0.90639 | 0.09361 |  |  |  |  |  |  |  |  |  |  |
