# Supplementary material for: Identification of candidate genomic regions for chicken egg number traits based on genome-wide association study
Source: BMC Genomics. 2021 Aug 10;22:610. doi: 10.1186/s12864-021-07755-3 (PMC8356427; doi:10.1186/s12864-021-07755-3)
Supplement: Supplementary file 3 — Additional file 3: Table S3. Results for comparison with previously reported QTLs. a The unit is Mb. U and D represent that SNP located upstream and downstream of the gene, respectively. b The unit is Mb. U and D represent that SNP located upstream and downstream of the gene, respectively. [file 12864_2021_7755_MOESM3_ESM.docx]

| Table S3 Results for comparion with previously reported QTLs. The results show that there are 4 QTLs overlapped with SNPs obtained from this study | | | | | | | | | | | |
| --- | --- | --- | --- | --- | --- | --- | --- | --- | --- | --- | --- |
| **Trait** | **Classification** | **Chromosome** | **Position** | **-log10 (P-value)** | **SNP ID** | **Candidate gene** | **Distance^a^** | **QTL** | **Distance^b^** |  |  |
| Egg production | Egg production rate | 13 | 16603463 | 6.09 | AX-75745363 | ***ENSGALG00000029896*** | within | 16604190-16604230 | 0.00073 |  |  |
|  | Small yellow follicle number | 4 | 68430908 | 5.39 | AX-76715084 | ***GRXCR1*** | U0.046 | 68473628-68473668 | 0.043 |  |  |
| Reproduction traits | Ovary weight | 7 | 25213170 | 5.26 | AX-77011457 | ***NA*** | NA | 66805-35685813 | within |  |  |
|  |  | 7 | 26516128 | 5.17 | AX-77014656 | ***SLC15A2*** | U0.023 | 66805-35685813 | within |  |  |
|  |  |  |  |  |  | ***IQCB1*** | D0.047 |  |  |  |  |
| ^a^ The unit is Mb. U and D represent that SNP located upstream and downstream of the gene, respectively. | | | | | | |  |  |  |  |  |
| ^b^ The unit is Mb. U and D represent that SNP located upstream and downstream of the gene, respectively. | | | | | | |  |  |  |  |  |
