# Supplementary material for: Identification of candidate genomic regions for chicken egg number traits based on genome-wide association study
Source: BMC Genomics. 2021 Aug 10;22:610. doi: 10.1186/s12864-021-07755-3 (PMC8356427; doi:10.1186/s12864-021-07755-3)
Supplement: Supplementary file 4 — Additional file 4: Figure S1. Admixture plot. Each color represents separate groups, each line represents a group value. [file 12864_2021_7755_MOESM4_ESM.docx]

| Figure S1. Admixture plot | | | | | | | | |
| --- | --- | --- | --- | --- | --- | --- | --- | --- |
| 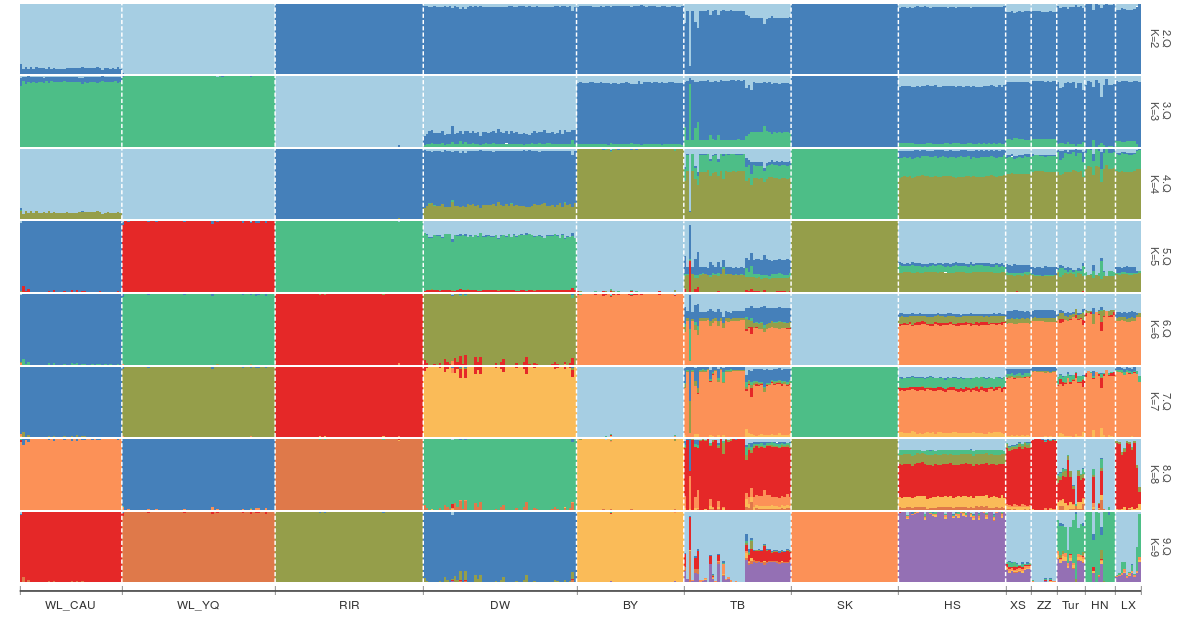   \|  \| \| --- \| |  |  |  |  |  |  |  |  |
|  |  |  |  |  |  |  |  |  |
|  |  |  |  |  |  |  |  |  |
|  |  |  |  |  |  |  |  |  |
|  |  |  |  |  |  |  |  |  |
|  |  |  |  |  |  |  |  |  |
|  |  |  |  |  |  |  |  |  |
|  |  |  |  |  |  |  |  |  |
|  |  |  |  |  |  |  |  |  |
|  |  |  |  |  |  |  |  |  |
|  |  |  |  |  |  |  |  |  |
|  |  |  |  |  |  |  |  |  |
|  |  |  |  |  |  |  |  |  |
|  |  |  |  |  |  |  |  |  |
|  |  |  |  |  |  |  |  |  |
|  |  |  |  |  |  |  |  |  |
|  |  |  |  |  |  |  |  |  |
| Each color represents separate groups, each line represents a group value. | | | | | | | | |
